# Addressing the Social Needs of Medicaid Enrollees Through Managed Care: Lessons and Promising Practices from the Field

**DOI:** 10.1089/pop.2021.0142

**Published:** 2022-02-02

**Authors:** Bettye A. Apenteng, Linda Kimsey, Samuel T. Opoku, Charles Owens, Angela H. Peden, William A. Mase

**Affiliations:** ^1^ Department of Health Policy and Community Health and Jiann-Ping Hsu College of Public Health, Georgia Southern University, Statesboro, Georgia, USA.; ^2^ Center for Public Health Practice and Research, Jiann-Ping Hsu College of Public Health, Georgia Southern University, Statesboro, Georgia, USA.

**Keywords:** social determinants of health, Medicaid, managed care, knowledge sharing, promising practices, lessons learned

## Abstract

With growing recognition of the adverse health impacts of unmet social needs, Medicaid managed care organizations (MMCOs) are increasingly focusing on addressing the social needs of Medicaid enrollees as part of a holistic approach to care. Information and knowledge sharing among MMCOs pertaining to lessons learned and promising practices from their social determinants of health (SDOH) targeted efforts can help identify successful practical approaches for navigating common challenges, developing robust SDOH programming, and effectively delivering whole-person care. Using data from interviews with 28 representatives of 8 national and regional MMCOs, this qualitative study describes the perspectives of MMCO representatives on the lessons learned and emerging promising practices from addressing SDOH among their Medicaid enrollees. Participants discussed the importance of member and community-centeredness, structured programming, and delivery system realignment in the effective delivery of whole person care. Ten lessons learned and emerging promising practices are discussed. Findings from this study suggest that success in addressing the social needs of Medicaid beneficiaries may be achieved through adaptive, data-driven, member- and community-centric efforts by MMCOs, facilitated by system-level changes that formally integrate social services within health care. Lessons learned and promising practices can serve as a foundation for identifying and evaluating best practices and guidelines for effective MMCOs' SDOH-related programming.

## Introduction

The World Health Organization^1^ defines social determinants of health (SDOH) as the nonmedical factors that significantly shape health outcomes, including the economic, sociocultural, political, and physical environmental contexts within which individuals are born, live, work, and grow.^1^ SDOH can adversely affect health functioning and outcomes by restricting access to health-promoting opportunities and resources.^2^ Existing evidence suggests that SDOH may wield a greater influence on an individual's health outcomes than medical care and lifestyle choices.^1^ Addressing SDOH is a vital means of correcting the health inequities that drive disparities in health care access and outcomes, more commonly experienced by vulnerable and underserved populations.^2,3^

As evidence of the health impacts of SDOH mounts, it has become increasingly clear that efforts to advance individual and population health must include a holistic approach that factors in both the medical and social needs of patients. Accordingly, there has been an increased focus on addressing the nonmedical needs of socially vulnerable populations, such as those enrolled in the Medicaid program. In an effort to improve health outcomes and lower costs in their respective Medicaid programs, states are leveraging the care coordination and preventive care capabilities of managed care organizations (MCOs) to address the nonmedical needs of Medicaid beneficiaries.^4,5^

Although Medicaid MCOs (MMCOs) recognize the value of addressing SDOH and have accepted their leadership role in this regard,^6^ research documents challenges that constrain their ongoing efforts, including regulatory-related constraints^7^ and difficulties in ascertaining programming effectiveness and return on investments.^8^ As MMCOs' role in addressing nonmedical needs continues to evolve and as they navigate expanded state expectations regarding this endeavor, MMCOs have expressed the need for more guidance.^6^ Knowledge sharing among MMCOs on the lessons learned and promising practices from their SDOH efforts can help identify successful practical approaches for navigating common challenges, developing robust SDOH programming, and effectively delivering whole-person care.

This study describes the perspectives of representatives of MMCOs on the lessons learned and emerging promising practices from addressing SDOH among their Medicaid enrollees. The study is particularly significant given the ongoing COVID-19 pandemic that has brought renewed attention to health inequities and the importance of addressing individuals' social needs.^9^

## Methods

Data for this descriptive qualitative study were obtained as part of a larger research study on MMCO efforts to address SDOH in eight states: Florida, Georgia, Hawaii, Kentucky, Illinois, New Jersey, New York, and South Carolina. Using purposive and snowballing sampling approaches, MMCOs serving these eight states were invited to participate in this study. Those agreeing to participate identified potential MMCO representatives whose work was most directly related to SDOH or who could provide insight on the subject from an organizational perspective. Some national plans connected the research team with leadership or teams from their individual state plans. Semi-structured interviews were conducted with 28 representatives of 14 unique MMCO markets across the eight Medicaid managed care states and Vermont. These MMCOs included three regional MCOs and state-specific markets of five national MCOs, including three of the largest five MCOs in the United States with respect to Medicaid managed care market share. MMCO representatives interviewed included senior plan executives, medical directors, directors of case management, directors of legal and regulatory affairs, and directors/staff of SDOH-focused departments or programs. Participants were asked to reflect on their organizations' efforts to address the social needs of Medicaid beneficiaries and share what worked well along with lessons learned from their successes and challenges.

Interviews were audio recorded and transcribed verbatim. Interviews lasted an hour, on average. Data were coded inductively and analyzed using thematic analysis following Braun and Clarke's^10^ approach. Two researchers independently coded the data, first immersing themselves in the data by reading and rereading the transcripts. A consensus codebook was then developed based on an initial coding of a subset of the transcripts and later was applied to all transcripts. Codes were then collated into emerging themes. Disagreements during the coding process were resolved through discussion and review by a third researcher, as necessary. This study was reviewed and approved by the Institutional Review Board at Georgia Southern University (#H20352).

## Results

Lessons learned and promising practices shared by participants fell under four themes: member-centeredness, community-centeredness, structured yet adaptable SDOH programming, and realigning systems for whole-person care.

### Member-centeredness

Drawing on their respective organizations' experiences in addressing the social needs of Medicaid enrollees (ie, members), participants stressed the importance of centering SDOH-related programming around member needs and preferences. Participants shared their organizations' success in implementing multipronged efforts to connect and intentionally engage Medicaid members in their care. Lessons learned and promising practices emerging from these discussions are highlighted next.

#### Member engagement is pivotal to SDOH programming. High-touch approaches increase connection to members

Participants noted that regardless of an MMCO's intent, efforts to care for members holistically are often futile if members cannot access or are unwilling to use the resources provided by MMCOs. Member engagement was thus identified as a prerequisite for effective SDOH-related programming. As one participant noted, “you can't help a member if you can't get in contact with a member.”

Approaches that increased contact and interaction with members were identified as effective ways to build member trust and increase member engagement. Participants stressed the importance of “meeting members where they are,” including leveraging care models that personalized the care delivery process, increased the number of touchpoints, and brought care to the patient. In-home visits by social workers and the use of community health workers were commonly cited approaches for increasing member engagement and facilitating the identification of member social needs.

“What puts us ahead of some people [is] we have people out there who are able to go out and actually connect with the member beyond the telephone, or beyond the computer, beyond anything else. I think it kind of shows the member that you are really connecting and really want to be involved in their care.”

“Well-trained community health workers who, in connecting [with members], drive that member engagement is key. And really, community health workers are kind of like the emerging [model] – you know – most of these people are not clinical by background but they know the communities well, they know the resources, they know people well and they're – again, they're kind of socially connected within our communities and that to me is the single greatest insight of how effective these people can be. You know, I'm a doctor, I work with nurses and things like that, but it doesn't have to be a health care person. But really, it's just about finding people that can connect and drive and get that member engagement. [That] is key.”

### Community-centeredness

In recognition of the impact of community-level factors on member health, efforts to address social needs of members were founded on a community-based approach to care. Participants emphasized the importance of tailored and responsive population-based programming that extends the capacity of health care delivery systems to deliver holistic care by connecting members to community social service resources. Effective ways to engage community stakeholders in the delivery of whole person care required partnerships forged through meaningful relationship building and active listening.

#### “It takes a village”: Relationships and partnerships are key

The complex interactions among SDOH in shaping health outcomes require cross-sectoral collaborative partnerships. Participants emphasized the importance of leveraging community partnerships to address the social needs of members and the communities they serve. Addressing members' unmet social needs was seen as being outside the expertise of MMCOs and clinical providers, necessitating partnerships with community-based organizations and social services agencies skilled in addressing those needs.

“I think that's what makes it work for us…We understand this work can't be done by one individual, by one stakeholder. It is so community driven. We joke when we [are] in the community [that] it takes a village. But it does.”

“Partnership. This cannot be done without partnerships. True health care delivery has lots of steps and involves lots of different elements and so for this to be successful, there has to be partnerships, both internally and externally, and the elimination of silos…so that we are all rowing in the same boat, rowing the same way, headed to the same destination. And we will learn lessons, nothing will be perfect, but at least we'll all learn them together.”

#### Active listening and a “boots on the ground,” community-embedded approach to SDOH programming improves the ability to meet community and member needs

According to participants, effective and responsive programs to address social needs required in-depth knowledge of community needs and resources. Such knowledge was only acquired by being embedded within the community and actively listening to community stakeholders.

“I don't think you can make assumptions [about] the needs of communities. You have to be invested and ingrained in the community to be able to really understand the needs.”

“Listening to our partners, listening to whomever it is that we are trying to find an answer, a solution, close a gap for. We come into this work as humble servants, if you will. We're looking to help. We don't know exactly what is wrong, but we're definitely going to work together to make sure that we find an answer. So, listening has been a fundamental and an ongoing learning experience for me.”

Most MMCOs described adopting a grassroots approach to addressing member social needs that included hiring from within the communities they served, connecting with members directly within their communities, and forging partnerships with local community-based organizations and social service agencies.

“When I go back and I talk to finance, and they're like, 'wait a minute. This guy was like $1 million a year. And we are down to basically nothing. What happened?'…Those are the times where I am able to go and say, 'This is why we need a high-touch approach. That is why we need to be in the field. That's why it doesn't need to be someone from Texas contacting the member'.”

“The community coalitions we stand on, it's really us being very much boots on the ground in the community, assessing community needs as well as community gaps.”

### Structured, yet adaptable programming

Participants described an intentional, structured, and coordinated approach to addressing members' social needs as a best practice that should be adopted by all MMCOs. By dedicating resources specifically to the social needs of members, MMCOs demonstrated a commitment to holistic care. However, participants cautioned that MMCOs must remain cognizant of the challenges in providing care for a vulnerable, transient population, often with complex medical and social needs. As one participant noted: “It takes time.” Reflecting on their organizations' efforts to address member social needs, participants shared lessons and promising practices for developing agile SDOH programming.

#### Dedicating a program and resources toward addressing SDOH at the organizational level is good practice

When asked to identify best practices and lessons learned from their organizations' efforts to address SDOH, a consistent theme among participants was the need for a structured and organized approach to addressing SDOH. They highlighted the value of dedicating specific resources to this endeavor, including having specific SDOH departments and staff within the organization:

“I think one of the best practices is having an organized program. A program that has a vision and strategies and goals that are set up to identify those members [with social needs].”

“I think having a program that is specifically dedicated to social determinants of health and not just as a piece of it is a best practice. Having people who are solely focused on that is important.”

#### It takes a special team of passionate advocates to advance work on SDOH

Participants noted that, because of the complexities associated with caring for Medicaid populations, work in this space was challenging. Effectively engaging members required staff members who were passionate and dedicated to advocating and caring for vulnerable populations. Addressing member needs also required staff (including nonclinical staff) with expertise in health and human services. Accordingly, several participants highlighted the need for selective hiring of an empathetic, transdisciplinary team with complementary skills in clinical and social services.

“It comes down to making sure that you hire someone who wants to do this. Because it's challenging. It's rewarding but it's challenging. And so, if you don't have the right person trying to help your members, you are not going to be successful.”

“I basically interviewed every person and said, this is my expectation. If you are joining my team, you cannot have any bias towards these individuals [Medicaid beneficiaries]. Our job is to serve them, and we try to meet them where they're at. If at any point, if you're brought on to the team, and I see that at any point there's a bias or any type of judgmental behavior, I will not hesitate to cut you loose from the team…I think my team is amazing. I have an amazing group…The team was built from the ground up and every single person on my team [is a] strong, passionate advocate for the population we serve. Really pounding the pavement and going out there to see how we can best serve them.”

#### Adaptable playbooks can provide evidence-based guidelines for designing and implementing SDOH related programming for Medicaid beneficiaries

In addition to dedicating specific resources to SDOH efforts and hiring appropriate staff, participants also highlighted the value of having established guidelines for developing SDOH programming. They emphasized the need for these to serve as frameworks that can be tailored to specific contexts, rather than a rigid approach to programming. National plan representatives discussed their success in developing and documenting “playbooks” for structured, yet adaptable programming across their markets based on lessons learned from pilot projects or from individual states' successes.

“And playbooks. Lots of playbooks. We're very big on playbooks. Once we learn, ooh, this was a really tough case, right, or this was a very tough situation, we always take time to debrief. Sit down collectively as a group, gather the information about what worked, what didn't work, how it worked, why it worked, what didn't work, why. Then, documenting it and saying okay, this didn't work because of the following. Is it something we can influence in the future? …There is no cookie cutter. We can try our best to have a playbook, we can try best to standardize workflows and processes, and to make it as easy.”

“They're [ie, corporate office] another one who is making playbooks and saying, here is the playbook. Here is exactly how to run this program or this project. Take and put whichever one best fits your state. Because you look at Florida and you look at Alabama, two totally different states. Yet, the model could work for both, but the programs within that model are gonna be totally different.”

#### Leveraging data and analytics to inform SDOH programming efforts improves responsiveness and effectiveness

Participants consistently discussed the importance of data in informing SDOH programming. As one participant described succinctly: “You need good data.” Data were described as vital for the assessment of social needs, the prioritization of efforts and for assessing the impact of efforts to address social needs. Participants noted that there remained opportunities for improvement with respect to assessing the impact of SDOH efforts and developing evidence-based guidelines and best practices.

Really, being able to do good research in terms of mapping out our state's population as whole as well as our membership population to determine what some of the common issues are, where some of the trouble spots are, and really taking a step back and looking at things in terms of the whole population, as opposed to smaller pockets…That's pretty huge.”

“You can't measure what you can't see. So, all the assessments we need to do. We need to do many more. We need a much more robust assessment strategy than we're trying to implement today, but once we get that information, I think we can better measure and see what's happening.”

#### Effective SDOH programming requires flexibility, agility, and a continuous improvement mindset

Agility and flexibility were identified as important attributes for MMCO SDOH activities because it allowed for MMCOs to adjust to, what was in some cases, a steep learning curve in the area of social services. As one participant noted, MMCOs venturing into SDOH work have to “[learn] to roll with the punches and ride that roller coaster and be ready for that adaptation.” Another expanded on this sentiment as follows:

“All of this work is innovative. All of this work is new. And so, we may have an expectation but if we cannot be flexible it is not going to be successful in the end because there are going to be things that we find out that we're going to have to tweak or add to the components or enhance. So, we've got to – because it's innovative we've got to remain flexible.”

Participants also noted that adoption of a continuous improvement mindset was vital to advancing efforts targeted at total person care. They mentioned that new and innovative ideas regarding SDOH programming often came from member-facing staff and those on the front lines of SDOH efforts.

“You hear that old saying, if it ain't broke, don't fix it. But for me, it's more of it ain't broke, how can we make it better for the next time? Let's continuously find ways to improve because all that is going to do is help our members. It's going to help our partners and it is going to help the state as a whole to become a healthier community.”

“I have meetings with teams across the state…and so, I always say to them…, 'if you see an issue, bring me a solution and we are going to talk about it.' They are the experts. They are the ones out there with members, and so that is where our best ideas have come from – from the team who has been in the field, who has found an issue and found that there is a resource lacking or came up with a better way.”

### Realigning systems for whole-person care

For participating MMCOs, their experiences in addressing SDOH had further emphasized the importance of having systems in place to support whole-person care. This included reconceptualizing the existing health care delivery system to integrate social services more effectively. Participants discussed the following specific lessons learned, with respect to system realignment:

#### There is a need for a paradigm shift in how we view health and deliver health care

According to participants, an effective transition to whole-person care required us to rethink health care delivery as we know it. An expanded view of health, to include social health, required a paradigm shift and an alteration of the status quo. Participants noted that champions were needed to mitigate potential resistance to this paradigm shift, and promote interdisciplinary and intersectoral collaboration to advance SDOH work.

“The thing is getting people to understand: what are social determinants of health? Why is it so incredibly important? Why should we have a department that works on that? I would say the number one barrier was really the paradigm shift. Just getting individuals to see it differently was a culture shift…You're changing the way people view things. Getting them to buy into that was just immense. It can be exhausting, but if you have strong champions and people who fully believe in what they're doing, they're believable.”

#### Innovative payment and delivery models for aligning medical and social services are needed for an effective transition to whole-person care

Participants stressed that delivering quality whole-person care required a systemic integration of social and medical services. Doing this effectively and efficiently would require the recognition of social services providers and community-based services as key players in health care. It also would require infrastructure to support data exchange and payment mechanisms across the health care and social services sectors. Some MMCOs participating in this interview noted that while they had already started moving in the direction of integrating social services into the health care ecosystem, more needs to be done in this regard.

“If we could figure out a way to really build in payment structure for those community-based organizations, I think that they would be more open to submitting those quote-on-quote claims or encounters that they have with our members.”

“Where you talk about sustainability and support, that's what it was about. It was about building those fundamental building blocks to create a sustainable environment and ecosystem for the CBO's [ie, community-based organizations] because now they are part of a clinical system basically.”

MMCOs noted that ultimately, efforts to address SDOH among the Medicaid population would be ineffective without strong state support. They described existing payment structures as a potential disincentive for SDOH programming and expressed the need for state-level policy reform focused on reimbursement for addressing member social needs.

“By looking at [the] rate setting. If you lower costs, then the states are reducing base rates so the incentive can be punitive. The question then shifts to, is the state willing to keep investment to allow MMCO to continue to innovate?”

“Just a broad look at the funding…In order to have true impact for Medicaid enrollees you really have to have a budget line item for social determinants. You just do. And so, having a policy that supports it across all enrollees in the state, [and] building it into a contract.”

The lessons and promising practices for MCO SDOH efforts are summarized in Table 1.

**Table 1.** Lessons and Promising Practices for Medicaid Managed Care Organizations' Social Determinants of Health Programming to Address the Social Needs of Medicaid Enrollees

Lessons	Promising practices
**Member-centeredness**	
1. Member engagement is pivotal to SDOH programming. High-touch approaches can increase member engagement.	Use approaches that allow MCOs to meet members where they are, including models such as community health worker and social worker in-home models that personalize the care delivery process, increase the number of touchpoints, and bring care to the patient.
**Community-centeredness**	
2. “*It takes a village”:* Relationships and partnerships are key.	Adopt a community-centered approach to programming that leverages community partnerships to address the social needs of members and the communities served.
3. Active listening and a “boots on the ground,” community-embedded approach to SDOH programming improves the ability to meet community and member needs.	Adopt a community-embedded approach to SDOH programming through local hiring and actively listening to community stakeholders.
**Structured, Yet Adaptable Programming**	
4. Dedicating a program and resources toward addressing SDOH at the organizational level is good practice.	Dedicate organizational resources specifically toward addressing SDOH, including having specific staff and departments responsible for SDOH programming.
5. It takes a special team of passionate advocates to advance work on SDOH.	Implement processes to ensure selective hiring of an empathetic, transdisciplinary team with complementary skills in clinical and social services.
6. Adaptable playbooks can provide evidence-based guidelines for designing and implementing SDOH-related programming for Medicaid beneficiaries.	Develop playbooks to serve as a framework for structured, yet adaptable programming across settings. Playbooks can be based on lessons learned from the challenges and successes of past SDOH programming efforts.
7. Leveraging data and analytics to inform SDOH programming efforts improves responsiveness and effectiveness.	Measure and track. Use data to determine social needs, to prioritize SDOH efforts, and to assess the impact of efforts to address SDOH.
8. Effective SDOH programming requires flexibility, agility, and a continuous improvement mindset.	Adopt an open-minded mindset and be willing to adapt, innovate, and evolve.
**Systems Realignment for Whole-Person Care**	
9. There is a need for a paradigm shift in how we view health and deliver health care.	Leverage internal organization champions to mitigate potential resistance to the paradigm shift to whole-person care, to promote interdisciplinary and intersectoral collaboration, and to advance SDOH work.
10. Innovative payment and delivery models for aligning medical and social services are needed for an effective transition to whole-person care.	Formally integrate social services into the health care ecosystem through investment in infrastructure to support data exchange and payment mechanisms across the health care and social services sectors.

MCO, managed care organization; SDOH, social determinants of health.

## Discussion

This study aimed to describe lessons learned from MMCO SDOH efforts that could serve as foundations for promising practices and structured efforts to address the social needs of Medicaid enrollees. Informed by their experiences, participants described a commitment to vulnerable populations, partnerships and relationship building, community-centeredness and embeddedness, flexibility, and agility as essential elements for effective SDOH programming. Intentional member engagement through high-touch approaches, the dedication of organizational resources to address specific member social needs, and the adoption of a data-driven approach to address social needs were identified as promising practices for facilitating SDOH efforts. In addition, participants noted that advancing SDOH efforts required a restructuring of existing health care delivery and financing models to facilitate effective integration of medical and social services. By reflecting on and sharing lessons learned from their successes, participants identified promising practices that could potentially serve as the foundation for the development of best practice guidelines.

Meaningful engagement with Medicaid beneficiaries was identified as a necessary foundation for effective whole-person care. The long-standing challenges MMCOs face in contacting and connecting with patients have been documented in other studies.^11–13^ MMCO representatives in this study recommended the use of models, such as the use of community health workers (CHWs), that enhance connection and contact with members. The CHW model has been associated with decreased cost and appropriate utilization of health care resources.^14^

The concept of community, including the importance of community-centered programming also was an emerging theme. Participants stressed the importance of tailoring SDOH programming to community needs and embedding SDOH efforts within existing community networks. In a recent study of Medicare MCOs' perspectives on their role in addressing SDOH, partnerships – particularly between MCOs and community-based social service organizations – emerged as an important instrument for driving MCO efforts to address the social needs of Medicare beneficiaries,^6^ a finding confirmed by the present study. Previous studies also have highlighted the importance of a multi-sectoral, “all-hands-on-deck” approach to addressing SDOH and advancing health equity.^15–17^

Data and analytics can serve as important catalysts for innovative, evidence-driven SDOH programming.^13,17^ Attempts at optimizing processes for such programming may come with challenges that participants noted would require open-mindedness as well as a willingness and ability to adapt, innovate, and evolve. Opportunities for sharing lessons learned and best practices in addressing SDOH can facilitate the diffusion of evidence-based, efficient, and effective SDOH-targeted interventions among MMCOs. However, as found in this study and supported by others,^18^ efforts aimed at improving whole-person care delivery may still be hampered without the restructuring of existing payment systems.

Knowledge sharing among MMCOs on the lessons learned and promising practices from their SDOH efforts are lacking. This study attempted to bridge this gap in the literature. However, as with all qualitative studies, the study is limited in the extent to which these findings can be generalized to MMCOs beyond those participating in the study. Future mixed methods research studies, especially those using nationally representative samples of MMCOs, are needed to deepen our understanding of MMCOs' role and impact on addressing the social needs of Medicaid enrollees, and to identify evidence-based practices for effective programming to address member social needs within managed care.

## Conclusion

Participants shared lessons learned in addressing the social needs of Medicaid beneficiaries enrolled in managed care that can be extended to other managed care settings, including Medicare and commercial insurance. They noted that efforts to address the social needs of enrollees must pivot on intentional member engagement and community-centeredness, be implemented systematically through tailored programming, and facilitated by innovative payment and health delivery models that promote the integration of social services into the existing medical model. Participants shared promising practices including the implementation of models, such as the CHW model that increased contact with members and facilitated member engagement and trust; the dedication of organizational resources specifically to addressing SDOH, and the implementation of nimble, community-embedded; and data-driven programming to enhance the delivery of whole-person care.

Taken together, the findings from this study suggest that success in addressing the social needs of Medicaid beneficiaries may be achieved through adaptive, data-driven, member- and community-centric efforts by MMCOs, facilitated by system-level changes that formally integrate social services within health care. Thus, MMCOs can incorporate these emerging promising practices into their efforts to provide holistic care. These efforts, however, cannot occur in a vacuum. There also is a need for state and federal policies to better align health care financing with the delivery of whole-person care and to facilitate the integration of medical and social services.
